# Imaging the attenuation coefficients of positron beams in matter: positron attenuation tomography

**DOI:** 10.1186/2197-7364-2-S1-A20

**Published:** 2015-05-18

**Authors:** Charles Watson

**Affiliations:** Siemens Healthcare, Knoxville, TN USA

A new positron annihilation imaging modality is described that enables nondestructive measurement of the linear attenuation coefficients (LACs) of positron beams in heterogeneous materials. This positron attenuation tomography (PAT) technique utilizes a positron emission tomography (PET) system embedded within a uniform static magnetic field, such as is found in integrated PET/MRI scanners. A Ga68-generated positron beam constrained by a 3T magnetic field penetrates objects placed within the scanner. The positrons slow down and annihilate within the object. The resulting annihilation distribution is tomographically imaged by the PET camera. This image may be interpreted as a map of the product of the positron beam's flux and its LAC at each point in the volume. It is shown that under certain easily achieved conditions this image can be decomposed into separate maps of the flux and the LACs, without need for auxiliary measurements. Although these LACs may depend on both beam and material properties, a beam softening correction is demonstrated that effectively removes the dependence on beam variation, leaving a relative LAC that is characteristic of the material. Unlike x-ray, gamma-ray or other transmission techniques, PAT does not require the penetration of the beam entirely through the object. High resolution and high contrast images of positron beam LACs in objects may be produced over nearly the full range of the positron beam, which for Ga68 beta-rays in a 3T field is about 0.5 g/cm^2^. The first examples of PAT images and an initial characterization of performance will be presented.

